# Preserving natural teeth versus extracting them: a willingness to pay analysis

**DOI:** 10.1186/s12903-022-02404-x

**Published:** 2022-09-05

**Authors:** Sulmaz Ghahramani, Nazanin Ziar, Najmeh Moradi, Kamran Bagheri Lankarani, Mohammad Sayari

**Affiliations:** 1 Health Policy Research Center, Institute of Health, Shiraz University of Medical Sciences, Shiraz, Iran; 2 Health Management and Economics Research Center, Health Management Institute, Iran University of Medical Sciences, Tehran, Iran

**Keywords:** Tooth, Preservation, Extraction, Willingness to pay analysis, Iran

## Abstract

**Background:**

Maximum willingness to pay (WTP) for a health benefit is related to perceived value. The goal of this study was to find out how much Iranian healthy people would be willing to pay to keep their natural teeth instead of having them pulled. This was done separately for the anterior and posterior teeth.

**Methods:**

The highest value was posed as an open-ended question in this cross-sectional analysis conducted in 2021. Four distinct scenarios for treating a tooth with a poor prognosis for natural tooth preservation versus extraction were offered. WTP for the preferred treatment option was asked for painful and painless anterior and posterior teeth separately. A two-stage hurdle approach was employed to determine factors influencing the WTP for a hopeless case. The level of significance was fixed at 0.05.

**Results:**

Out of 795 individuals, 355 (44.7%) were male and 209 (26.3%) had poor self-stated dental health. Over 65% of those interviewed said they wanted to keep their teeth. The mean WTP was highest for dental preservation up to 94 USD and the lowest was for extraction without replacement 19 USD. The WTP for anterior tooth therapy was greater than the WTP for posterior dental care, regardless of treatment type or tooth discomfort. Participants with higher education, jobs, income-to-expenditure matching, older age, preference for the treatment in a private office, and female gender (except for WTP for a painful posterior tooth) were more likely to have a WTP of at least 1 USD.

**Conclusion:**

The average WTP for treatment of teeth with a poor prognosis was lower than the average fee charged in dental facilities, and more than 65% of participants preferred to keep their teeth. Regardless of the treatment option or whether it was painful or not, WTP for anterior teeth treatment was higher than for posterior teeth. Generally, we found that sociodemographic factors influenced WTP decision-making the most. This study has practical implications for public oral health policymakers and insurance organizations.

**Supplementary Information:**

The online version contains supplementary material available at 10.1186/s12903-022-02404-x.

## Introduction

Dental decay is one of the most prevalent non-communicable diseases on a global scale, but its treatment is costly accounting for 5–10% of the healthcare expenditure in industrialized countries and is one of the leading causes of hospitalization for children in some high-income countries [1]. Individual oral hygiene, perceptions of dental visits, not knowing what the dentist will perform, and the expense of treatment may all predict less frequent dental visits or postponement of dental checkups or dental treatments (dental avoidance) [2]. Many people put off dental visits for financial reasons as well; hence, the economic cost of treatment plays a significant role in influencing a patient's capacity to get dental care [3]. On the other hand, patients' access to oral health care is negatively affected by the large and ongoing rise in the cost of providing care.

As is the case with many other outpatient clinical disciplines, patients with hopeless teeth should be involved in treatment selection and decision-making regarding dental therapy achieved. On the other hand, public approval and agreement will be contingent upon their assessment of the procedure. Additionally, the patient's assessment is a significant factor in the decision-making process [4]. When multiple alternatives with varying costs are presented to an individual, if the relative benefits of one service are seen to be larger than the incurred expenses, the individual may purchase that service; and Willingness-to-pay (WTP) is a frequently used metric for calculating the monetary value of health benefits and for determining differences in preferences among related health care choices [5]. WTP is a useful economic metric in dentistry since it enables meaningful comparisons across diverse healthcare settings. Its underlying premise is that the maximum price individuals are prepared to pay for a health benefit is proportional to their perceived value of that benefit.

There is less research on how dental treatment expenses for decayed, hopeless teeth affect the usage of services and therapy desired by healthy individuals.

The degree of tooth decay ranges from a slight discoloration to the destruction of the tooth's crown, and it can spread to the roots. Early treatment can reduce tooth loss and preserve natural teeth [6]. In cases of pulp involvement and significant crown destruction, endodontic therapy is often required and other accessible treatments vary from simple amalgam and composite buildup to partial and complete crown positioning [6]. Among many conditions for a hopeless tooth, we chose the presence of a hopeless tooth and the treatment options are to preserve or to extract; each option has pros and cons [7, 8]. The purpose of this study was to determine the willingness of Iranian adults to pay for a hopeless tooth that could be treated in alternative hypothetical scenarios (natural tooth preservation versus extraction scenarios) and to investigate associated factors. The findings will be useful for oral health policymakers and insurers.

In this study, healthy adults’ WTP was investigated because people typically visit dentists when they experience pain and their condition becomes complicated, making treatment more difficult and expensive, which may affect patients' treatment choices based on cost, complications, and duration [9]. Additionally, to our knowledge, there is limited information regarding Iranian's WTP for natural tooth preservation.

## Materials and methods

### Setting and sample

In this cross-sectional study, we used an online questionnaire that was widely shared between April and March 2021. The method tried to be representative of the normal population of Iran in terms of gender, age, education level, and employment status. The study population included individuals who were not under treatment by a dentist at that time, which may have affected their WTP.

The method of sampling was convenient. Inclusion criteria for the study were that the participants were at least 18 years old and willing to participate in the study.

### Data collection

The ethics committee of Shiraz University of Medical Sciences accepted this study, and the ethical code is IR.SUMS.DENTAL.REC.1399.083. After obtaining ethical and administrative approval, a questionnaire was created using the online questionnaire platform (PORSLINE). At the outset, it was explained that participation was voluntary, the questionnaire was anonymous, and a phone number was provided in case of ambiguity or queries.

### Questionnaire design

The questionnaire was divided into three sections. To begin, broad demographic information was gathered, as well as income and expenditure, habits and oral hygiene, and history of dental issues. Additionally, the clinic to which they prefer to refer for dental treatments was collected, such as a public clinic, a private clinic, or a private office; their use of insurance and the type of insurance / complimentary insurance; and their history of dental pain in the previous month were also collected. Due to the poor response rate for family income and expenses [10], we chose to inquire about "matching revenues and expenses" as a proxy for the family's economic position.

Second, we could not directly examine the oral health of included participants due to the COVID-19 crisis and diversely distributed samples. Instead, we employed a self-reported 11-item questionnaire that was shown to have good psychometric properties for measuring the state of oral health compared to the oral health status that was identified by an exam of a dentist and an anesthesiologist [11]. According to the instruction, the raw scores should be converted to a 0–100 linear scale after the logit transform. The cut-off value was set at 52 points for measuring the state of oral health. An increase in self-reported oral health score means worse oral health.

Finally, individuals were asked to imagine that they have a decaying tooth with a deep cavity that requires treatment. And then, to facilitate comprehension, the following four treatment choices were illustrated:

1. Preservation of a natural tooth with root canal therapy, crown lengthening surgery, and crown positioning
2. Tooth extraction and implant placement
3. Tooth extraction and placement of a fixed partial denture (bridge)
4. Tooth extraction without replacement

It was declared that the treatment strategies require considerable effort on the part of both the therapist and the patient, and have a very dubious prognosis. The key distinctions between the four recommended remedies were discussed, as well as the benefits and drawbacks of four distinct alternative treatments were described in detail [12–14]. (Additional file 1). After doing a pilot study with 20 people to test the study questionnaire, we found that it is better to add pictures of the four therapy options (Additional file 1) in the study.

Afterward, through the online platform, four hypothetical scenarios were presented for anterior painful, anterior painless, posterior painful, and posterior painless situations, consecutively. It was explained that their WTP for the preferred hypothetical scenario only applies to one decayed tooth.

Then, individuals were asked whether they would be ready to pay for a hypothetical clinical condition associated with the existence of a hopeless tooth. Subjects who indicated they would prefer a treatment were asked “which” treatment option they preferred, and finally, they were asked to define the maximum amount they would be willing to pay for dental care as an open-ended value, to determine the expenditure deemed appropriate by the patient for their choice.

Individual valuations were determined by WTP in terms of the out-of-pocket costs associated with receiving the intervention in the absence of insurance coverage. The order of the painful or painless, and anterior or posterior scenarios had no impact on the WTP nor the proportion of zero responses.

### Statistical analysis

A two-part hurdle procedure was used in this study to ascertain the major parameters influencing WTP for treatment of hopeless teeth [15]. This approach is capable of dealing with extremely skewed count data with an excess of zeroes [16]. The first section employed logistic regression to estimate the chance of WTP for the treatment of a hopeless tooth. Due to the dispersion of data in the second portion, negative binomial regression was used to determine the components associated with positive WTP. Additionally, marginal effects were estimated to quantify the effect of a slight change in predictors on the result [17, 18]. Multivariate logistic regression was also used to look at the relationships between sociodemographic factors and self-reported dental health.

The level of significance was fixed at 0.05. To reduce potential biases associated with extremely high WTP values, the highest centile of each response variable is excluded from the analysis [19]. After removing 11 extreme outliers from the 806 cases, the analysis was conducted on 795 individuals. Due to the considerable volatility of the currency rate, we used the simple moving average over the last 200 days to convert values to US dollars. As a result, the US dollar is regarded to be equal to 230,000 IRR.

## Results

### The data set

Around 1000 Iranians viewed the online questionnaire, and 900 responded to the questions. Among the 806 instances that responded entirely, 11 extreme outliers were excluded from the data set, leaving 795 individuals for study. As independent variables, the data set comprises age, sex, marital status, education, employment status, basic insurance, residence, self-reported questionnaire score (self-reported oral health), and revenue and spending matching. Additionally, we evaluated the WTP for a painless and painful anterior tooth and the WTP for a painless and painful posterior tooth as dependent variables.. In the main data, excluding the outliers, the dependent variable of WTP comprised the amount stated by the participants who preferred one of the treatment options and then were willing to pay for it (hence, zero responses were not included in the calculations).

Table 1 summarizes the subgroups of qualitative variables, their frequencies, and the descriptive statistics for quantitative variables. Mean and SD of WTPs for main data includes all WTP amounts including zero responses. As seen in Table 1, the participants' mean WTP for anterior teeth was greater than their WTP for posterior teeth. There were a total of 209 individuals with poor self-reported oral health (26.3%). Additionally, as expected, people were willing to pay a premium for painful teeth over non-painful teeth. Table 2 illustrates the frequency of desired therapy for four different categories of hopeless teeth.

**Table 1:** The frequency of qualitative variables and common descriptive statistics for quantitative variables (N = 795)

Qualitative variables				
Variable	Subgroups	Frequency	Percent	
Gender	Male	355	44.7	
	Female	440	55.3	
Marital status	Single	311	39.1	
	Married	438	55.1	
	Divorced	27	3.4	
	Widow	19	2.4	
Education	Associate or lower	266	33.5	
	Bachelor or higher	529	66.5	
Employment status	Unemployed	150	18.9	
	Employed	410	51.6	
	Retired	67	8.4	
	Job seeker	61	7.7	
	Housewife	107	13.5	
Domicile	Metropolis	390	49.1	
	Non-metropolis	405	50.9	
Basic insurance	Don't have	185	23.3	
	Have	610	76.7	
Matching between revenues and expenses	No	479	60.3	
	Yes	316	39.7	
Self-report oral health	Poor	209	26.3	
	Good/fair	586	73.7	
The dentist you prefer for treatment of dental problem	Specialist dentist	423	53.2	
	General dentist	58	7.3	
	Not important	314	39.5	
Center you prefer for treatment of dental problem	Private office	309	38.9	
	Private clinic	124	15.6	
	Public clinic	86	10.8	
	Not important	276	34.7	

Quantitative variables				
Variable	Minimum	Maximum	Mean*	SD*
Age	18	83	35.90	13.43
self-reported questionnaire score (0–100 linear measure)	0	69.19	46.77	8.75
WTP for painless anterior	0	870	82.16	127.91
WTP for painful anterior tooth	0	870	87.68	127.11
WTP for painless posterior tooth	0	870	64.70	99.84
WTP for painful posterior tooth	0	870	69.86	105.00
Total WTP (for painless anterior and posterior and painful anterior and posterior tooth)	0	3480	304.40	418.70

*WTP* Willingness to pay

*Mean and SD of WTPs for main data including zero responses

**Table 2:** The frequency of preferred treatment and their associated WTP

	Painless anterior tooth			Painful anterior tooth			Painless posterior tooth			Painful posterior tooth		
	Frequency	Percent	Mean WTP	Frequency	Percent	Mean WTP	Frequency	Percent	Mean WTP	Frequency	Percent	Mean WTP
RCT + CL + Crown	618	77.7	87.50	613	77.1	93.64	524	65.9	76.86	523	65.8	83.18
Extraction and implant	104	13.1	82.38	104	13.1	89.76	101	12.7	72.73	112	14.1	71.28
Extraction and bridge	50	6.3	52.96	46	5.8	51.61	65	8.2	49.00	69	8.7	53.57
Extraction without replacement	23	2.9	1.04	32	4.0	18.66	105	13.2	5.99	91	11.4	3.88

RCT + CL + Crown: Root canal therapy, crown lengthening surgery and crown positioning

In almost all scenarios, participants with painful teeth had more WTP than those with painless teeth, the maximum mean ± SD of WTP for main data belonged to WTP for a painful anterior tooth (87.68 ± 127.11). The results were that three participants had less WTP for a painful anterior tooth, which will be treated with extraction and bridge, than painless anterior ones; six and four participants had less WTP for a painful posterior tooth, which will be treated with extraction and implant and extraction without replacement, respectively.

### Zero responses

Tables 3 and 4 indicate the frequency of qualitative factors and common descriptive statistics for quantitative variables for participants who had zero responses. In Additional file 1: Table S4, the frequency of zero response participants for different treatment options is detailed. The zero response participants involve both the participants who do not prefer being treated so were unwilling to pay and the participants who prefer the treatment but were unwilling to pay for it. Only one participant for posterior painful and two for posterior painless scenario preferred the treatment but were unwilling to pay for it. All the zero response respondents who stated a cause for it pointed to the financial barrier for this decision.

**Table 3:** The frequency of qualitative variables for zero response participants

Variable	Subgroups	Painless anterior tooth		Painful anterior tooth		Painless posterior tooth		Painful posterior tooth	
		Frequency	Percent	Frequency	Percent	Frequency	Percent	Frequency	Percent
Gender	Male	60	57.1	55	55.6	93	52.8	72	50.7
	Female	45	42.9	44	44.4	83	47.2	70	49.3
Marital status	Single	45	42.9	43	43.4	65	36.9	53	37.3
	Married	51	48.6	49	49.5	98	55.7	77	54.2
	Divorced	4	3.8	4	4.0	6	3.4	5	3.5
	Widow	5	4.8	3	3.0	7	4.0	7	4.9
Education	Associate and less	60	57.2	57	57.6	89	50.6	74	52.2
	Bachelor and higher	45	42.8	42	42.4	87	49.4	68	47.8
Employment status	Unemployed	37	35.2	33	33.3	47	26.7	39	27.5
	Employed	34	32.4	35	35.4	69	39.2	48	33.8
	Retired	9	8.6	8	8.1	16	9.1	14	9.9
	Job seeker	13	12.4	10	10.1	17	9.7	18	12.7
	Housewife	12	11.4	13	13.1	27	15.3	23	16.2
Domicile	Metropolis	49	46.7	43	43.4	77	43.8	65	45.8
	Non-metropolis	56	53.3	56	56.6	99	56.3	77	54.2
Basic insurance	Don't have	31	29.5	31	31.3	49	27.8	41	28.9
	Have	74	70.5	68	68.7	127	72.2	101	71.1
Matching between revenues and expenses	No	83	79.0	78	78.8	138	78.4	108	76.1
	Yes	22	21.0	21	21.2	38	21.6	34	23.9

**Table 4:** The descriptive statistics of quantitative variables for zero response participants

	Variable	Minimum	Maximum	Mean	SD
Painless anterior tooth	Age	18.00	76.00	33.44	13.93
	Self-reported questionnaire score	28.89	69.19	49.95	7.83
Painful anterior tooth	Age	18.00	78.00	34.02	14.53
	Self-reported questionnaire score	33.57	69.19	50.03	7.67
Painless posterior tooth	Age	18.00	78.00	35.11	13.69
	Self-reported questionnaire score	33.57	69.19	50.03	7.56
Painful posterior tooth	Age	18.00	78.00	35.53	14.52
	Self-reported questionnaire score	33.57	69.19	50.50	7.85

### Two-part hurdle model

The two-part hurdle model was performed using the twopm command in Stata 16.0 [20]. Table 5 summarizes the results of the two-part hurdle model in terms of odds ratio and incidence rate ratio for the logit and negative binomial parts, respectively. The findings show that individuals with bachelor's degrees or higher had a greater probability of WTP of at least 1 USD in all scenarios as compared to those with associate's degrees or lower. Additionally, those whose income matched their expenditures had a higher probability of WTP. Employed participants had a higher probability of WTP than unemployed people. Additionally, an increase in the self-reported oral health score is associated with a decrease in the WTP value. In all cases except WTP for a painful posterior tooth, females had a higher probability of WTP of at least 1 USD compared to males. Increases in self-reported oral health scores are associated with a decrease in the probability of WTP in all situations. In all of the scenarios, people were willing to pay less in general clinics than in offices.

**Table 5:** The results of the two-part hurdle model

Variable (reference group)	WTP for painless anterior tooth		WTP for painful anterior tooth		WTP for painless posterior tooth		WTP for painful posterior tooth	
	Logit part	NB part	Logit part	NB part	Logit part	NB part	Logit part	NB part
Gender (male)								
Female	2.21*	0.89	1.84*	1.01	1.59*	1.01	1.46	1.03
Marital status (single)								
Married	1.00	0.95	1.29	0.97	0.93	0.84	0.95	0.91
Divorced	0.49	0.96	0.62	1.06	0.67	0.96	0.76	1.04
Widow	0.42	0.94	1.17	0.88	0.61	0.82	0.51	0.91
Education (associate and less)								
Bachelor and higher	2.27*	1.06	2.37*	1.12	1.73*	1.30*	1.71*	1.11
Employment status (unemployed)								
Employed	3.22*	1.21	2.39*	1.17	2.00*	1.37*	2.43*	1.57*
Retired	2.01	1.23	1.80	1.02	1.42	1.24	1.65	1.43
Job seeker	0.90	1.14	1.10	1.09	0.95	1.26	0.68	1.44
Housewife	1.83	0.93	1.40	0.76	1.13	0.97	1.20	0.99
Basic insurance (don't have)								
Have	0.83	1.08	1.03	0.95	0.92	0.80*	0.97	1.03
Domicile (metropolis)								
Metropolis	0.78	1.15	0.97	1.07	0.97	1.12	0.83	1.11
Matching between revenues and expenses (no)								
Yes	2.35*	1.57*	2.25*	1.40*	2.48*	1.44*	1.99*	1.51*
Dentist (specialist)								
General	0.79	1.52*	0.68	1.03	0.61	1.26	0.51	1.08
Each	0.64	0.99	0.72	1.01	0.64*	1.08	0.70	1.00
Center treatment (office)								
Private clinic	0.40*	0.76*	0.55*	0.68*	0.52*	0.92	0.56	0.81
Public clinic	0.77	0.37*	1.10*	0.46*	0.85	0.45*	0.75	0.50*
Each	1.07	0.70*	1.15*	0.70*	0.96	0.83	1.11	0.76*
Age	1.02	1.01	1.01	1.01*	1.01	1.02*	1.01	1.01*
Self-reported oral health	0.96*	0.96*	0.97*	0.97*	0.96*	0.97*	0.95*	0.97*

*Significant at the .05 level; NB negative binomial; WTP willingness to pay

### Marginal effects

Table 6 illustrates the marginal effects. The results reveal that individuals whose income matched their expenditures considered at least 32.94 USD more WTP than others in all scenarios. Employed respondents indicated a WTP of at least 20.94 USD more than unemployed respondents. Increased self-reported oral health scores are anticipated to result in a WTP reduction of at least 2.14 USD. Additionally, it is projected that adding one year of age increases the WTP by at least 0.74 USD. Participants valued their care in public clinics at least 41.55 USD less than treatment in offices.

**Table 6:** The results of marginal effect

Variable (reference group)	WTP for painless anterior tooth		WTP for painful anterior tooth		WTP for painless posterior tooth		WTP for painful posterior tooth	
	dy/dx	*P*-value	dy/dx	*P*-value	dy/dx	*P*-value	dy/dx	*P*-value
Gender (male)								
Female	− 3.94	0.632	5.69	0.5	5.21	0.438	5.24	0.457
Marital status (single)								
Married	− 4.57	0.622	− 0.55	0.954	− 12.42	0.124	− 6.93	0.394
Divorced	− 8.92	0.64	0.72	0.973	− 7.34	0.684	0.35	0.985
Widow	− 12.90	0.6	− 9.65	0.69	− 17.77	0.382	− 12.77	0.565
Education (associate and less)								
Bachelor and higher	10.82	0.178	16.78	0.035	21.00	0	11.97	0.079
Employment status (unemployed)								
Employed	24.10	0.013	20.94	0.049	25.95	0.001	35.77	0
Retired	22.46	0.204	6.77	0.702	15.78	0.261	25.74	0.082
Job seeking	8.44	0.542	7.72	0.613	12.18	0.29	15.88	0.185
Housewife	− 0.18	0.988	− 16.55	0.168	− 0.22	0.982	1.13	0.9
Basic insurance (don't have)								
Have	5.40	0.505	− 3.96	0.667	− 15.94	0.055	1.62	0.827
Domicile (metropolis)								
Metropolis	9.95	0.16	5.96	0.422	6.98	0.236	6.11	0.319
Matching between revenues and expenses (no)								
Yes	44.07	0	36.01	0	32.94	0	34.95	0
Dentist (specialist)								
General	40.62	0.061	− 0.43	0.979	10.70	0.461	− 0.94	0.946
Each	− 3.39	0.656	− 1.72	0.837	0.76	0.905	− 2.51	0.706
Center treatment (office)								
Private clinic	− 32.48	0.002	− 40.00	0	− 13.19	0.145	− 20.81	0.022
Public clinic	− 67.40	0	− 59.13	0	− 41.55	0	− 43.62	0
Each	− 30.98	0.001	− 31.76	0.001	− 13.03	0.074	− 19.03	0.013
Age	0.74	0.029	0.78	0.038	1.09	0	0.89	0.006
Self-reported oral health	− 3.24	0	− 2.96	0	− 2.14	0	− 2.61	0

*WTP* Willingness to pay

### Outlier data

To detect the effect of outlier data, the results of descriptive analysis and the two-part hurdle model are for all data, including outliers represented in Additional file 1: Tables S1–S3. This analysis showed that outliers affected the maximum WTP and marginal values, although the impact on the two-part hurdle model was minor.

In Additional file 1: Table S1, mean and SD of WTPs for all data (including outliers) belonged to amounts of the WTP response and zero responses. The maximum mean ± SD of WTP of all data including outlier was the WTP for painless posterior tooth (177.25 ± 2164.45). Frequency and mean ± SD WTP for different treatment options was separated for outliers and main data, with and without zero responses in Additional file 1: Table S4.

### Multivariate logistic regression

The result of multivariate logistic regression and the associated Odds Ratio (OR) is illustrated in Table 7. Female participants, compared to males, had significantly lower odds of poor self-stated dental health (OR = 0.5). Married and widowed participants compared to single ones had a higher likelihood of poor self-reported dental health. Participants with bachelor's and higher degrees compared to those with associate degrees and less were less likely to have poor self-reported dental health (OR = 0.432). The odds of having poor self-reported dental health in individuals living in metropolises were significantly lower than in those living in non-metropolises (OR = 0.579).

**Table 7:** The results of robust multivariate logistic regression for self-reported dental health

Variables (reference)	Odds ratio	SE	*P*-value	95% Confidence interval	
Gender (male)					
Female	0.500	0.106	0.001	0.330	0.757
Marital status (single)					
Married	1.666	0.407	0.037	1.032	2.689
Divorced	1.682	0.833	0.294	0.637	4.442
Widow	4.772	2.961	0.012	1.414	16.100
Education (associate and less)					
Bachelor and higher	0.432	0.080	0.000	0.300	0.623
Employment status (unemployed)					
Employed	0.627	0.174	0.093	0.364	1.081
Retired	1.331	0.556	0.494	0.586	3.020
Job seeker	1.052	0.402	0.894	0.498	2.225
Housewife	1.042	0.359	0.905	0.531	2.045
Basic insurance (don't have)					
Have	1.216	0.270	0.378	0.787	1.880
Domicile (non-metropolis)					
Metropolis	0.579	0.105	0.003	0.405	0.826
Matching between revenues and expenses (no)					
Yes	0.797	0.151	0.232	0.550	1.156
Age	1.011	0.009	0.201	0.994	1.029

## Discussion

The purpose of this study was to ascertain the WTP for natural tooth preservation versus extraction in the Iranian population, separately for anterior and posterior teeth. The mean WTP for dental caries management varied between 1 and 94 USD depending on the treatment method. The mean WTP for treatment of a tooth with a poor prognosis was lower than the average fee charged at dental facilities, which is consistent with a lower WTP for tooth filling services when compared to the actual charges imposed on a population with limited restorative services [21]. Additionally, this study's findings indicate that the whole WTP for dental care for a tooth with a poor prognosis is a small percentage of the GDP per capita of 2282.5 USD, as determined by the World Bank (https://data.worldbank.org/). There are studies on WTP for various health outcomes in Iran [10, 22–24], but studies are either too limited in the field of dentistry to compare with current research findings or too focused on the characteristics of WTP associated with dental disorders. For example, in Iran, the WTP for an orthodontic treatment cycle was 20 million Rials [25].

Regardless of the treatment option or whether the tooth was painful or not, the WTP for anterior tooth treatment was greater than the WTP for posterior dental treatment. Even though root canal therapy for posterior teeth is more expensive than for anterior teeth due to the greater number of canals and the higher overall treatment cost of posterior teeth, participants' WTP for anterior teeth was greater than for posterior teeth, indicating that aesthetics and appearance may be more important than mastication for some populations [9]. Even though these claims need more research in different populations and cultures, they were not supported by data from Tanzanian patients, who were about as willing to pay (WTP) to have their back teeth filled or pulled as they were to have their front teeth filled or pulled [21].

The results indicated that more than 65% of participants favored tooth preservation over tooth extraction, with or without substitutes. While the frequency of such decisions varied according to the anterior or posterior tooth, it appears that patients favor more conservative treatment approaches that result in tooth lay-up [21, 26].

This research found that the WTP for treating painful teeth was higher than for treating painless teeth due to the discomfort caused. Dental decay that results in a hole or crack in their tooth and causes food retention or irritates the tongue or mucosa may result in a referral to a dentist, although the patient may refuse treatment if offered root canal therapy of a decayed painless tooth and may reply, “I'm not in pain, so it's fine.”

The study population's self-reported oral health score was low. Similarly, in all scenarios, those with zero responses had worse self-reported oral health. Around 26% of individuals had poor self-reported dental health, which should be further studied in terms of its association with an increased risk of caries. Unsatisfactory oral health behavior among diverse population groups in Iran has been demonstrated previously [27], but there is still a dearth of population-based studies employing standard instruments for oral health assessment [28]. Only 15% of respondents in Tehran, Iran's capital city, reported having poor oral health when asked to characterize their current oral health [29]. This discrepancy could be explained in part by the fact that our research population was diverse and came from all around Iran, whereas this study was conducted in Tehran, a huge metropolis city. Second, the discrepancy could be explained by the fact that participants in our study graded their dental health using a standard checklist vs. a single item in the Tehran survey cited above. This investigation indicated various percentages of zero responses. The most frequently occurring scenario for zero response was a painless posterior tooth (more than 22%), while the least frequently occurring scenario was a painful anterior tooth (12.5%). Additionally, refusal to pay for a hopeless tooth was more likely to occur in males, those with a lower level of education, those with a mismatch between revenues and expenses, and residents of smaller cities. In general, our findings reveal that sociodemographic parameters are the most influential elements influencing WTP decision-making. These results back up the study's conclusion that the choice of WTP is based on some factors, including the patient's income, the setting of the clinic, and their gender [9].

Participants with bachelor's or higher degrees compared to those with associates or lower degrees, working participants compared to jobless people, and participants whose income matched their expenditures all had a greater probability of having a WTP of at least 1 US dollar. This condition, which includes educated participants who are employed and earn an acceptable salary, results in a participant's economic position and ability to pay to increase in all scenarios. As can be shown from the marginal analysis, participants with an appropriate income level and employed participants had significantly higher WTP for at least 32 and 20 USD more than others in all situations. In all cases except WTP for a painful posterior tooth, females had a higher likelihood of WTP of at least 1 USD compared to males. It has been said that the WTP for oral health interventions changes for women, people with more education, and people with more money [9, 30, 31].

Additionally, when participants' ages increased, the value of WTP also increased. This goes against previous research [21, 31] that showed that younger people were more willing to pay for dental health interventions.

Participants who preferred in-office treatment had a higher probability of WTP of at least one dollar compared to those who preferred private or public clinics. The setting of oral service for treatment of teeth with a poor prognosis also showed a significant marginal effect, such that participants considered paying at least 41.55 USD less for treatment in public clinics than in private offices. In other locations, the therapeutic setting was also a significant factor in WTP [9, 32].

Participants of this study with worse self-reported oral health scores had less WTP for treatment of a poor prognosis tooth. This finding is contrary to intuition. For instance, in Italy, patients with regular once-or-twice-a-year dental checkups agreed to pay additional money for the choice of treatment for decayed teeth [26]. In Finland, healthy people with no subjective need for dental care had a greater WTP for immediate treatment of a lost filling [33]. Regression analysis, to better elucidate this paradox, showed that self-reported dental health varies with socio-demographic characteristics including gender, education, and domicile.

For the first time in Iran, this study conducted a national-level analysis of the WTP for natural tooth preservation versus extraction using a hypothetical scenario. The findings have practical consequences for both policymakers and insurance companies. Although this study was constrained by the nature of the online questionnaires employed, it did demonstrate the likely influence of a pandemic on WTP values. It is also worth noting that the context in which WTP questionnaires are constructed has an impact on the outcomes [34, 35]. In this study, we used ex-post contexts for the WTP scenarios in which individuals assume they have a clinical condition and they have to pay out of pocket, and so the ability to pay will have an impact on the WTP. As a substitute, in the ex-ante context, respondents pay for insurance, and the ability to pay does not have such a strong impact on the WTP. Because we only used one method to figure out WTP, and there are many others, more research using other methods to figure out WTP for oral health interventions may be needed to back up our findings.

## Conclusion

The average WTP for treatment of teeth with a poor prognosis was lower than the average fee charged in dental facilities, and more than 65% of participants preferred to keep their teeth. Regardless of the treatment option or whether it was painful or not, WTP for anterior teeth treatment was higher than for posterior teeth. Generally, we found that sociodemographic factors influenced WTP decision-making the most. This study has practical implications for public oral health policymakers and insurance organizations.

## Supplementary Information

**Additional file 1.** Supplementary Results.

## Data Availability

All data generated or analyzed during this study is included in this published article and its Additional file 1.
