# Supplementary material for: Preserving natural teeth versus extracting them: a willingness to pay analysis
Source: BMC Oral Health. 2022 Sep 5;22:375. doi: 10.1186/s12903-022-02404-x (PMC9441316; doi:10.1186/s12903-022-02404-x)
Supplement: Supplementary file 1 — Additional file 1. Supplementary Results. [file 12903_2022_2404_MOESM1_ESM.docx]

**Supplementary appendix**

**Supplement to:** Preserving natural teeth vs. extracting them: a willingness to pay analysis

Table S1. The frequency of qualitative variables and common descriptive statistics for quantitative variables including outlier data (N=806).

| **qualitative variables** | | | | | |
| --- | --- | --- | --- | --- | --- |
| **Variable** | **Subgroups** | **Frequency** | | **Percent** | |
| Gender | Male | 361 | | 44.8 | |
|  | Female | 445 | | 55.2 | |
| Marital status | single | 315 | | 39.1 | |
|  | married | 444 | | 55.1 | |
|  | divorced | 28 | | 3.5 | |
|  | widow | 19 | | 2.4 | |
| Education | associate or lower | 271 | | 33.6 | |
|  | bachelor or higher | 535 | | 66.4 | |
| Employment status | unemployed | 154 | | 19.1 | |
|  | employed | 413 | | 51.2 | |
|  | retired | 70 | | 8.7 | |
|  | job seeker | 61 | | 7.6 | |
|  | housewife | 108 | | 13.4 | |
| Domicile | metropolis | 399 | | 49.5 | |
|  | Non-metropolis | 407 | | 50.5 | |
| Basic insurance | don't have | 186 | | 23.1 | |
|  | have | 620 | | 76.9 | |
| Matching between revenues and expenses | no | 484 | | 60.0 | |
|  | yes | 322 | | 40.0 | |
| Self-report oral health | Poor | 210 | | 26.1 | |
|  | Good/fair | 596 | | 73.9 | |
| Dentist you prefer for treatment of dental problem | Specialist dentist | 429 | | 53.2 | |
|  | General dentist | 58 | | 7.2 | |
|  | Not important | 319 | | 39.6 | |
| Center you prefer for treatment of dental problem | Private office | 314 | | 39.0 | |
|  | Private clinic | 126 | | 15.6 | |
|  | Public clinic | 86 | | 10.7 | |
|  | Not important | 280 | | 34.7 | |
| **quantitative variables** | | | | | |
| Variable | Minimum | | Maximum | Mean^*^ | Std. Deviation^*^ |
| age | 18.00 | | 83.00 | 35.92 | 13.47 |
| self-report questionnaire score (0-100 linear measure) | 0.00 | | 69.19 | 46.72 | 8.74 |
| WTP for painless anterior | 0.00 | | 4348.00 | 94.36 | 212.76 |
| WTP for painful anterior tooth | 0.00 | | 13043.00 | 134.55 | 682.21 |
| WTP for painless posterior tooth | 0.00 | | 43478.00 | 177.25 | 2164.45 |
| WTP for painful posterior tooth | 0.00 | | 43478.00 | 130.94 | 1534.66 |
| Total WTP (for painless anterior and posterior and painful anterior and posterior tooth) | 0.00 | | 87434.00 | 537.10 | 3567.09 |

WTP willingness to pay

* Mean and Std. deviation of WTPs for all data including zero responses.

Table S2. The results of two-part hurdle model including outlier data.

| Variable (Reference group) | WTP for painless anterior tooth | | WTP for painful anterior tooth | | WTP for painless posterior tooth | | WTP for painful posterior tooth | |
| --- | --- | --- | --- | --- | --- | --- | --- | --- |
|  | Logit part | NB part | Logit part | NB part | Logit part | NB part | Logit part | NB part |
| Gender (Male) |  |  |  |  |  |  |  |  |
| Female | 2.22* | 0.90 | 1.84* | 0.81* | 1.61* | 0.93 | 1.45 | 0.68* |
|  |  |  |  |  |  |  |  |  |
| Marital status (single) |  |  |  |  |  |  |  |  |
| Married | 1.09 | 0.90 | 1.40 | 0.96 | 0.92 | 0.78* | 0.97 | 0.63* |
| Divorced | 0.58 | 1.29 | 0.72 | 1.27 | 0.69 | 0.50* | 0.78 | 0.77 |
| Widow | 0.50 | 0.75 | 1.37 | 0.67 | 0.59 | 0.34* | 0.51 | 0.49 |
|  |  |  |  |  |  |  |  |  |
| Education (associate and less) |  |  |  |  |  |  |  |  |
| bachelor and higher | 2.11* | 0.77* | 2.20* | 0.90 | 1.63* | 1.24 | 1.67* | 0.91 |
|  |  |  |  |  |  |  |  |  |
| Employment status (unemployed) |  |  |  |  |  |  |  |  |
| Employed | 3.08* | 1.07 | 2.30* | 0.53* | 2.06* | 1.77* | 2.35* | 2.24* |
| Retired | 1.83 | 1.41 | 1.64 | 0.95 | 1.61 | 1.34 | 1.71 | 3.09* |
| Job seeker | 0.88 | 0.95 | 1.08 | 0.49* | 0.96 | 1.18 | 0.66 | 1.29 |
| Housewife | 1.67 | 0.79 | 1.29 | 0.39* | 1.15 | 0.75 | 1.16 | 1.76* |
|  |  |  |  |  |  |  |  |  |
| Basic insurance (don't have) |  |  |  |  |  |  |  |  |
| Have | 0.86 | 1.22* | 1.06 | 1.30* | 0.93 | 0.47* | 0.99 | 0.60* |
|  |  |  |  |  |  |  |  |  |
| Domicile (metropolis) | 1.00 | 1.00 | 1.00 | 1.00 | 1.00 | 1.00 | 1.00 | 1.00 |
| Metropolis | 0.77 | 1.39* | 0.95 | 1.31 | 1.00 | 1.76* | 0.85 | 1.60* |
|  |  |  |  |  |  |  |  |  |
| matching between revenues and expenses (no) |  |  |  |  |  |  |  |  |
| Yes | 2.22* | 1.53* | 2.12* | 1.30* | 2.47* | 2.24* | 1.98* | 1.97* |
|  |  |  |  |  |  |  |  |  |
| Dentist (specialist) |  |  |  |  |  |  |  |  |
| General | 0.83 | 1.42* | 0.72 | 0.95 | 0.61 | 1.35 | 0.51 | 1.12 |
| each | 0.62 | 0.98 | 0.69 | 0.92 | 0.65* | 2.14* | 0.70 | 1.18 |
|  |  |  |  |  |  |  |  |  |
| Center treatment (office) |  |  |  |  |  |  |  |  |
| Private clinic | 0.40* | 1.04 | 0.55* | 0.64* | 0.54* | 0.74* | 0.57 | 0.89 |
| Public clinic | 0.68 | 0.34* | 0.96 | 0.38* | 0.86 | 0.34* | 0.73 | 0.45* |
| each | 1.06 | 0.72* | 1.15 | 0.65* | 0.98 | 0.64* | 1.11 | 0.91 |
|  |  |  |  |  |  |  |  |  |
| Age | 1.02 | 1.01 | 1.01 | 1.00 | 1.01 | 1.03* | 1.01 | 1.00 |
| Self-report oral health | 0.96* | 0.96* | 0.96* | 0.95* | 0.96* | 0.99* | 0.95* | 0.97* |

*Significant at the .05 level; NB negative binomial; WTP willingness to pay

Table S3. The results of marginal effect including outlier data.

| Variable (Reference group) | WTP for painless anterior tooth | | WTP for painful anterior tooth | | WTP for painless posterior tooth | | WTP for painful posterior tooth | |
| --- | --- | --- | --- | --- | --- | --- | --- | --- |
|  | dy/dx | P-value | dy/dx | P-value | dy/dx | P-value | dy/dx | P-value |
| Gender (Male) |  |  |  |  |  |  |  |  |
| Female | -3.52 | 0.714 | -18.66 | 0.14 | -0.69 | 0.962 | -38.00 | 0.002 |
|  |  |  |  |  |  |  |  |  |
| Marital status (single) |  |  |  |  |  |  |  |  |
| Married | -9.49 | 0.379 | -0.66 | 0.962 | -35.82 | 0.052 | -52.47 | 0 |
| Divorced | 21.80 | 0.448 | 27.99 | 0.436 | -82.62 | 0 | -34.99 | 0.186 |
| Widow | -31.04 | 0.197 | -38.42 | 0.172 | -108.45 | 0 | -77.98 | 0.002 |
|  |  |  |  |  |  |  |  |  |
| Education (associate and less) |  |  |  |  |  |  |  |  |
| bachelor and higher | -19.08 | 0.083 | -3.10 | 0.813 | 34.40 | 0.007 | -3.43 | 0.779 |
|  |  |  |  |  |  |  |  |  |
| Employment status (unemployed) |  |  |  |  |  |  |  |  |
| Employed | 16.45 | 0.188 | -84.10 | 0.001 | 81.21 | 0 | 78.63 | 0 |
| Retired | 43.94 | 0.085 | -0.58 | 0.989 | 37.59 | 0.16 | 122.32 | 0 |
| Job seeking | -5.90 | 0.709 | -99.32 | 0 | 13.97 | 0.457 | 10.11 | 0.407 |
| Housewife | -13.62 | 0.34 | -117.44 | 0 | -19.31 | 0.165 | 42.31 | 0.004 |
|  |  |  |  |  |  |  |  |  |
| Basic insurance (don't have) |  |  |  |  |  |  |  |  |
| Have | 17.03 | 0.051 | 31.15 | 0.006 | -124.07 | 0 | -64.83 | 0 |
|  |  |  |  |  |  |  |  |  |
| Domicile (metropolis) |  |  |  |  |  |  |  |  |
| Metropolis | 28.88 | 0 | 33.17 | 0.002 | 69.81 | 0 | 47.84 | 0 |
|  |  |  |  |  |  |  |  |  |
| matching between revenues and expenses (no) |  |  |  |  |  |  |  |  |
| Yes | 48.06 | 0 | 42.63 | 0 | 121.35 | 0 | 81.94 | 0 |
|  |  |  |  |  |  |  |  |  |
| Dentist (specialist) |  |  |  |  |  |  |  |  |
| General | 38.52 | 0.107 | -10.48 | 0.645 | 24.08 | 0.298 | 2.76 | 0.894 |
| each | -6.09 | 0.499 | -14.59 | 0.238 | 94.49 | 0 | 13.66 | 0.23 |
|  |  |  |  |  |  |  |  |  |
| Center treatment (office) |  |  |  |  |  |  |  |  |
| Private clinic | -7.72 | 0.587 | -65.89 | 0 | -57.05 | 0.002 | -21.17 | 0.122 |
| Public clinic | -76.64 | 0 | -101.62 | 0 | -114.60 | 0 | -67.59 | 0 |
| each | -32.01 | 0.001 | -56.26 | 0 | -62.08 | 0 | -9.82 | 0.447 |
|  |  |  |  |  |  |  |  |  |
| Age | 0.68 | 0.089 | 0.24 | 0.658 | 3.49 | 0 | 0.48 | 0.351 |
| Self-report oral health | -4.16 | 0 | -6.28 | 0 | -2.38 | 0.005 | -4.18 | 0 |

WTP willingness to pay

Table S4: Frequency and mean ± Std. deviation of willingness to pay for different treatment options, for outliers and main data, with and without zero responses, separately

|  |  |  | Painless anterior tooth | Painful anterior tooth | Painless posterior tooth | Painful posterior tooth |
| --- | --- | --- | --- | --- | --- | --- |
| WTP including outlier | With zero | Number | 806 | 806 | 806 | 806 |
|  |  | Mean ± Std. Deviation | 94.36 (212.76) | 134.55 (682.21) | 177.25 (2164.45) | 130.94 (1534.66) |
|  | Without zero | Number | 700 | 706 | 629 | 664 |
|  |  | Mean ± Std. Deviation | 108.65 (224.89) | 153.61 (726.97) | 227.13 (2448.24) | 158.94 (1689.72) |
| WTP without outlier (main data) | With zero | Number | 795 | 795 | 795 | 795 |
|  |  | Mean ± Std. Deviation | 82.16 (127.91) | 87.68 (127.11) | 64.7 (99.84) | 69.86 (105.00) |
|  | Without zero | Number | 690 | 696 | 619 | 653 |
|  |  | Mean ± Std. Deviation | 94.66 (132.92) | 100.16 (106.18) | 83.10 (131.18) | 85.05 (110.15) |

**Explaining the feature**

Dental caries may have varying degrees of severity, ranging from a change in the colour of the tooth to the involvement of the dental pulp. In this study, we imagine situations for the anterior or posterior, painful or painless, hopeless tooth. This hopeless case could be treated with four alternative approaches. One is the preservation of this tooth, and three other situations are related to dental extraction.

1. Do you like to know about dental caries treatment outcomes and their advantages and disadvantages?
2. I know about treatments. Continue the questions.
3. I want to read more.

If you choose (b), the treatment description with images will be opened.

**Tooth extraction:**

A tooth extraction is a procedure to remove a tooth from the gum socket with or without surgery under local anesthesia. After the tooth extraction, the underneath bone starts to resorb and leads to bone loss. Further dental implant and prosthesis treatment may be complicated if the replacement of an extracted tooth takes a long time. So natural tooth preservation is important for keeping alveolar bone. When back teeth are lost and not replaced, the position of the teeth next to and opposite them can change.

After tooth extraction with or without surgery, you can face pain, swelling, and bleeding. Medication may be required depending on the case.


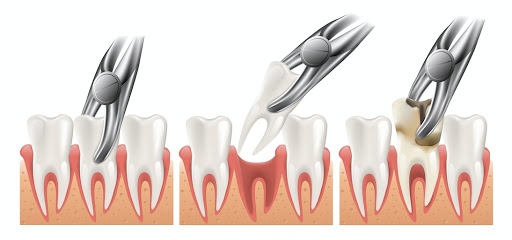


**Dental Implant:**

Nowadays, dental implants are one good treatment for tooth replacement. Dental implants can be inserted in sockets just after tooth extraction, or after a couple of weeks, or thereafter. The first steps are inserting the dental implant and bone grafting if needed. After abutment placement, an impression is taken and, in another visit, cementation of the crown will be done. The number of visits depends on the implant system and the dentist’s decision.

In cases where alveolar bone is not good enough for positioning the implant or when the sinus floor extends into the implant site, further surgeries are required. The insertion of implants keeps the bone around the implant strong and reduces bone resorb. Dental implants are a durable and successful treatment for the replacement of extracted teeth. Pain, swelling, and ecchymosis due to the surgical process may be seen, and instruction and avoidance will be given to the patient after surgery.

**
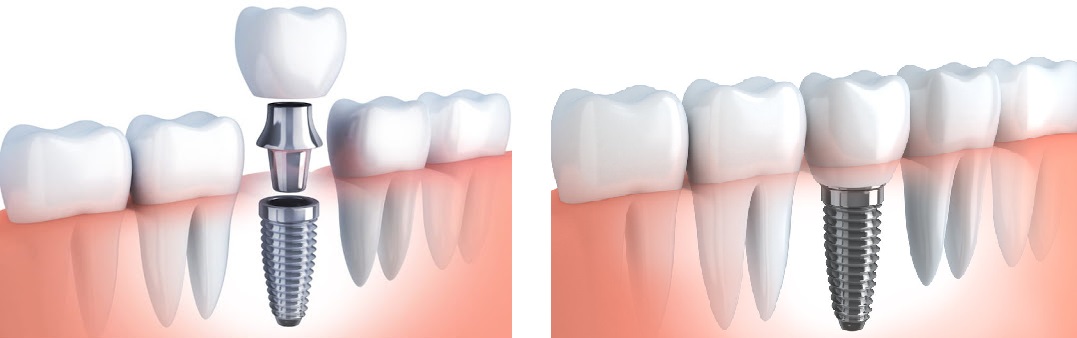
**

**Root canal therapy**

Root canal therapy is the process of removing unhealthy tissues from the tooth canals and subsequent cleaning and shaping of them, then obstruction of the decontaminated canals will be performed by material. Restoration of the tooth that has undergone root canal therapy can be completed simply or may require complicated restorative procedures. In severely destroyed teeth, crown positioning may be needed after filling. It can be done in a single or two visits.

After root canal treatment, pain may occur for 0–3 days and be relieved with analgesics.

**
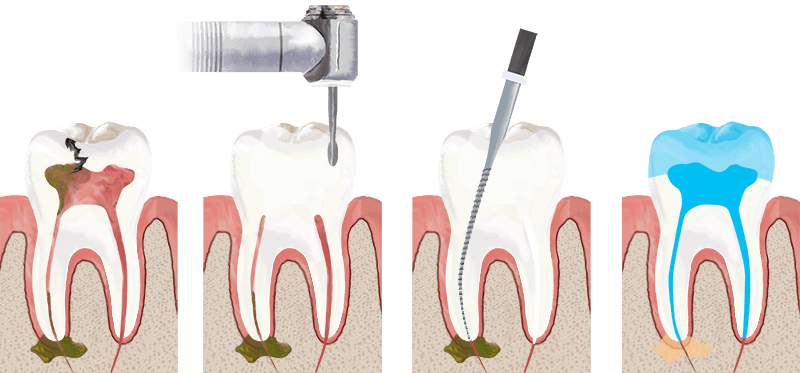
**

**Crown lengthening surgery**

Crown lengthening (CL) is a surgical procedure in which the gingiva and bone surrounding a tooth are removed for better support of fillings or a crown. Crown positioning needs intact dental tissue around the tooth for better support, so CL becomes essential if there isn’t sufficient tooth in place to hold the crown by itself. In some cases, CL can enhance the aesthetic and, in some cases, may cause a worse appearance. The complications are the same as other surgical complications.

**
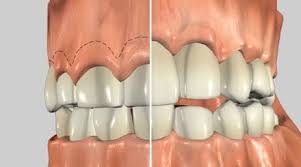
**

**Fix partial denture or bridge**

To replace an extracted tooth, the crown preparation will be done for the teeth on both sides of the extracted tooth. After the impression, the extracted tooth will be replaced by a pontic that is fused to the abutment teeth. The disadvantage of this treatment is the bone loss of the extracted tooth under the fixed partial denture and also the damage to the neighboring tooth for abutment preparation. It can, in some cases, sensitize the prepared teeth. Cleaning under the pontic with additional oral hygienic instruments should be done. The dental bridge‘s cost is lower than a dental implant, and the complications after surgery were not seen in this treatment.

**
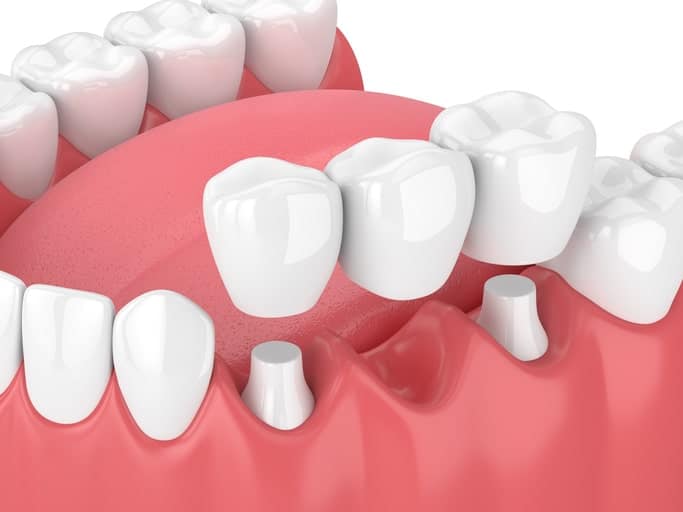
**

Note: These descriptions are abstract and correspond to our survey’s scenario. If you have had or will have the same situation, you should consult with your dentist to make a decision on your treatment. Every treatment for a decayed tooth may have varying complications, discomfort, and a recovery period.

*Continue with the scenarios…*

**Willingness to pay for dental services:**

It is important to imagine that there is only **ONE** hopeless tooth that should be treated and not all situations at the same time.

Imagine one of your... teeth that appear/doesn’t appear in your smile has severe dental caries and it is painful... The dentist proposes the following four treatments, and you can choose one of them. Regarding advantages and disadvantages, which treatment do you prefer?

1. Preservation of a natural tooth with root canal therapy, crown lengthening surgery, and crown positioning
2. Tooth extraction and implant replacement
3. Tooth extraction and bridge replacement
4. Tooth extraction without replacement

Are you willing to pay for the treatment of this tooth?

1. No (then the reason is asked)
2. Yes

Imagine you don’t have any basic or complimentary insurance and you have to pay by yourself. Regarding your revenue and savings, respond to the question, and if you don’t have income and savings, imagine that you can request a loan or borrow money. How much would you be willing to pay to receive dental care? (Maximum amount of money in Toman)
